# Uncovering transcriptomic landscape alterations of CAN-2409 in *in vitro* and *in vivo* glioma models

**DOI:** 10.3389/fmed.2023.1140352

**Published:** 2023-05-09

**Authors:** Marilin S. Koch, Mykola Zdioruk, Michal O. Nowicki, Michael S. Hoetker, Zachary T. Herbert, Francesca Barone, Paul P. Tak, E. Antonio Chiocca, Ghazaleh Tabatabai, Sean E. Lawler

**Affiliations:** ^1^Harvey Cushing Neurooncology Research Laboratories, Department of Neurosurgery, Brigham and Women’s Hospital, Harvard Medical School, Boston, MA, United States; ^2^Department of Molecular Biology, Massachusetts General Hospital, Boston, MA, United States; ^3^Molecular Biology Core Facilities, Dana-Farber Cancer Institute, Boston, MA, United States; ^4^Candel Therapeutics, Needham, MA, United States; ^5^Department of Neurology and Interdisciplinary Neuro-Oncology, University Hospital Tübingen, Hertie Institute for Clinical Brain Research, Eberhard Karls University Tübingen, Tübingen, Germany; ^6^Department of Pathology and Laboratory Medicine, Legorreta Cancer Center, Brown University, Providence, RI, United States

**Keywords:** transcriptomics, CAN-2409, oncolytic virotherapy, glioma, oncoimmunology

## Abstract

**Rationale:**

CAN-2409 is a locally delivered oncolytic therapy, which results in vaccination against the injected tumor. CAN-2409 consists of a non-replicating adenovirus armed with the Herpes virus thymidine kinase, which metabolizes ganciclovir into a phosphorylated nucleotide that is incorporated into the tumor cell’s genome, thereby inflicting immunogenic cancer cell death. While CAN-2409’s immunological impact has been well characterized, its effects on the tumor cells transcriptome remains unknown. We compared the transcriptomic landscape after treatment of glioblastoma models with CAN-2409 *in vitro* and *in vivo* to assess how the interplay with the tumor microenvironment influences CAN-2409-mediated transcriptome alterations.

**Methods:**

We performed RNA-Seq with CAN-2409 treated patient-derived glioma stem-like cells and tumors of C57/BL6 mice and compared KEGG pathway usage and differential gene expression focusing on immune cell and cytokine profiles. *T*-cell -killing assays were performed to assess candidate effectors.

**Results:**

PCA analysis showed distinct clustering of control and CAN-2409 samples under both conditions. KEGG pathway analysis revealed significant enrichment for p53 signaling and cell cycle pathway, with similar dynamics for key regulators of both pathways *in vitro* and *in vivo*, including *MYC, CCNB1, PLK1 and CDC20*. Selected alterations (PLK1 and CCNB1) were validated at the protein level. Cytokine expression analysis revealed upregulation of pro-inflammatory *IL12a* under both conditions; immune cell gene profiling showed reduction of myeloid associated genes. *T*-cell-killing assays showed increased killing in the presence of IL-12.

**Conclusion:**

CAN-2409 significantly alters the transcriptome both *in vitro* and *in vivo*. Comparison of pathway enrichment revealed mutual and differential utilization of pathways under both conditions, suggesting a modulating influence on the cell cycle in tumor cells, and of the tumor microenvironment on the transcriptome *in vivo*. IL-12 synthesis likely depends on interactions with the tumor microenvironment, and it facilitates CAN-2409 cell killing. This dataset provides potential to understand resistance mechanisms and identify potential biomarkers for future studies.

## 1. Introduction

Glioblastoma is the most frequent malignant brain tumor with an incidence of 3.19/100,000 in the United States (1). Despite extensive research activity and clinical trials in the recent years, mass debulking surgery followed by concomitant radiochemotherapy with temozolomide (2) remains standard of care. CAN-2409 is a non-replicating adenovirus expressing the Herpes simplex virus-thymidine kinase (tk). When administered together with a prodrug (acyclovir, ganciclovir or valacyclovir), CAN-2409 catalyzes its phosphorylation into “false” nucleotides that become incorporated into tumor cell DNA during repair or mitosis, eventually causing immunogenic tumor cell death associated with an influx of CD3^+^ *T*-cells (3). CAN-2409 has been shown to significantly prolong median overall survival compared to SoC in a phase II study for primary glioblastoma (4). A recent clinical trial of CAN-2409 combined with nivolumab demonstrated the induction of a systemic immune response after CAN-2409 administration to the resection bed (NCT03576612,) (5). CAN-2409 inflicts changes upon the transcriptome through incorporation of a toxic nucleotide, but these have not yet been evaluated for assessment of further therapeutic implications. The aim of this work was to characterize the impact of CAN-2409 on the transcriptome using *in vitro* and *in vivo* models of glioblastoma both to identify therapeutically relevant alterations with a focus on how the interplay between CAN-2409 and the tumor microenvironment further contributes to transcriptomic modifications.

## 2. Materials and methods

### 2.1. Cell culture

*In vitro* experiments were performed with the glioblastoma stem cell like cell (GSC) line G9_pCDH (6). The cell line was either cultured with Neurobasal Medium (Life technologies), supplemented with B27 (Invitrogen), 1% Glutamax (Invitrogen), 20 ng/mL EGF (Peprotech), 20 ng/mL FGF (Peprotech), Primocin (Invivogen) and Plasmocin (Invivogen) or Dulbecco’s modified Eagle’s Medium (DMEM) (Life technologies), supplemented with 10% heat-inactivated fetal bovine serum, Plasmocin and Primocin (both Invivogen). For *in vivo* experiments GL261fluc cells were utilized and cultured with DMEM as mentioned above. All cell lines were incubated at 37°C and 5% CO_2_ and tested for mycoplasma infection on a regular basis.

### 2.2. Reagents

For *in vitro* experiments, cells were treated with CAN-2409 (MOI 100) and 10 μg/mL ganciclovir, for *in vivo* experiments, 3 μL of AdV-tk with 2 × 10^8^ vector particles [vp]/μL were injected intracranially and animals treated with 20 mg/kg bodyweight ganciclovir intraperitoneally 2x/day for 7 days. Both AdV-tk and ganciclovir were kindly provided by Candel Therapeutics Inc. (Needham, MA, United States).

### 2.3. Animal experiments

For generation of *in vivo* samples, 100,000 GL261fluc cells were injected intracranially in 2 μL HBSS 2 mm right lateral, 1 mm frontal to the bregma, and 3 mm deep in the brains of albino C57/BL6 mice. Tumor growth was monitored by bioluminescence imaging with the Perkin-Elmer IVIS Lumina 3 after intraperitoneal of injection of D-Luciferin (#LUCK-1G, Gold Biotechnology).

14 days post-tumor implantation, CAN-2409 (treatment group) or HBSS (control) was stereotactically injected intratumorally by, followed by 7 days treatment with ganciclovir in the treatment group as described above. All animal experiments were performed according to BWH Center for Comparative Medicine IACUC guidelines. Animals were euthanized on the third day after end of therapy.

### 2.4. RNA isolation

For generation of *in vitro* samples, 500,000 G9_pCDH cells were seeded in triplicates and treated as mentioned before. For generation of *in vivo* samples, tumors, respectively, normal brain of the corresponding brain region were excised. RNA of both *in vitro* and *in vivo* samples was isolated with the RNeasy mini kit (#74104, Qiagen). RNA concentration was measured with NanoDrop and quality verified using an Agilent Bioanalyzer. 3 *in vivo* RNA samples per group were used for further analyses.

### 2.5. RNA Seq

Libraries were prepared using Roche Kapa mRNA HyperPrep sample preparation kits from 100 ng of purified total RNA according to the manufacturer’s protocol. The finished dsDNA libraries were quantified by Qubit fluorometer, Agilent TapeStation 2,200, and RT-qPCR using the Kapa Biosystems library quantification kit according to manufacturer’s protocols. Uniquely indexed libraries were pooled in equimolar ratios and sequenced on an Illumina NextSeq500 runs with single-end 75 bp reads by the Dana-Farber Cancer Institute Molecular Biology Core Facilities. Sequenced reads were aligned to the UCSC hg19 reference genome assembly and gene counts were quantified using STAR (v2.5.1b) (7). Differential gene expression testing was performed by DESeq2 (v1.10.1) (8) and normalized read counts (FPKM) were calculated using cufflinks (v2.2.1) (9). RNAseq analysis was performed using the VIPER snakemake pipeline (10).

### 2.6. Immunoblots

Lysates of G9pCDH cells and G98 cells were generated with 8 M urea buffer (8 M urea, 1% Triton 100, 50 mM Tris-base ph = 7.5, 100 mM NaCl, 1 mM EGTA, 1 mM RDTA, 50 mM beta-glycerophosphate), supplemented with protease inhibitors (Millipore-Sigma #539134) and phosphatase inhibitors (Millipore-Sigma/Roche #4906845001) after treatment with CAN-2409, AdVtk (MOI 500) or mock for 48 h. Protein concentration was determined with Bradford assay. Primary antibodies used were anti-CCNB1 (Cell Signaling Technologies #12231), anti-PLK1 (Cell Signaling Technologies, #4513), anti-c-MYC (Cell Signaling Technologies #5605) and Alexa Fluor 488-labeled anti-GAPDH (#sc-365,062, Santa Cruz), anti-rabbit (Cell Signaling Technologies #7074) was used a as secondary antibody. Band signal intensity quantification was carried out with Bio-Rad Image Lab software in triple measurements.

### 2.7. T-cell killing assays ± recombinant human IL-12

Human CD8^+^-T-cells were isolated from healthy blood donors’ PBMCs with Ficoll-Paque PLUS (GE Healthcare) and human CD8^+^-T-Cell Isolation Kit (Miltenyi Biotec) and subsequently activated with Dynabeads™ Human T-Activator CD3/CD28 for T-Cell Expansion and Activation (Gibco) (ratio 1:1) and human IL-2 (Peprotech) (10 ng/mL). G9_pCDH cells were seeded in a density of 750 cell/well in ultra-low-attachment 96-well plates (Corning). CAN-2409 and/or CD8^+^-T-cells together with human recombinant IL-12 (R&D Systems) were added 48 h later. Spheres were incubated for 6 days further with daily acquisition of microscope images (Nikon TI, magnification 4x). Sphere area was assessed with ImageJ.

### 2.8. Statistics

Further statistical analysis of RNA Seq data was performed using *R*. Analysis of *T*-cell killing assays was performed with unpaired t-tests in Graph Pad Prism (version 9).

### 2.9. Graphics

Illustrations were generated with BioRender (publication agreements numbers OS24UGAKQ0, MS24UGAGGH, TI24UGABY0).

## 3. Results

### 3.1. CAN-2409 is associated with downregulation of transcription associated pathways *in vitro* and *in vivo*

**Figure 1:**
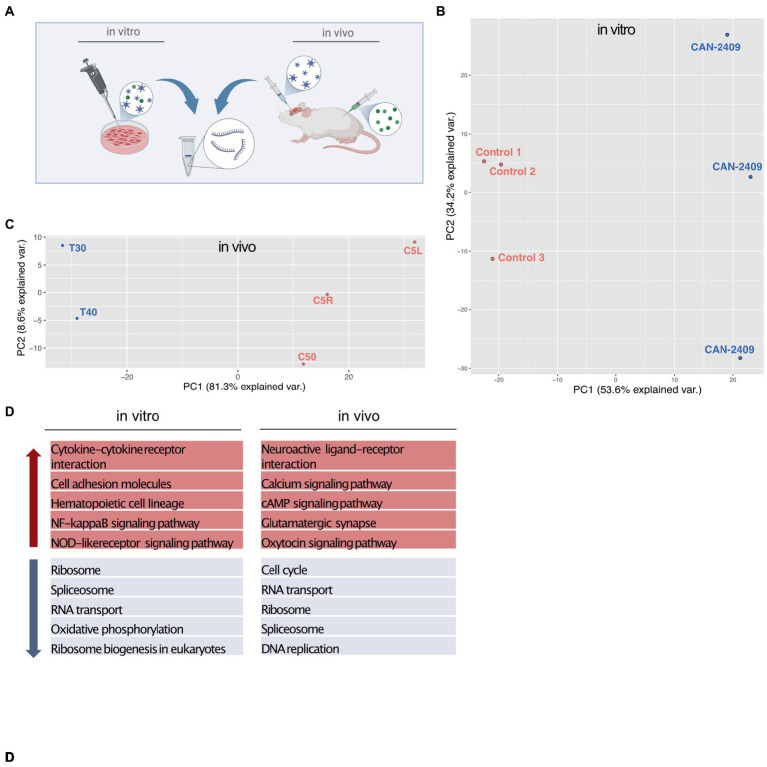
**(A)** Experimental setup *In vitro* samples were isolated from G9_pCDH glioma cells after 72 h incubation time with CAN-2409. *In vivo* samples were generated from brains of albino C57/BL6 mice 3 days after treatment with CAN-2409. **(B,C)** PCA analysis *in vitro* and *in vivo* both sample sets display spatial segregation depending on the treatment, explaining 87.8% of sample clustering *in vitro* and 89.9% *in vivo*. D TOP 5 KEGG pathways *in vitro* and *in vivo* Up (red arrow) and down (blue arrow) regulated pathways *in vitro* and *in vivo*.

To assess the effect of CAN-2409 on the glioblastoma cell transcriptome, we isolated total RNA after treatment *in vitro* and *in vivo* and performed RNA-Seq and compared with mock treated controls (Figure 1A). Initial PCA analysis observed an outlier in the *in vivo* sample batch, that was excluded for further analyses (Supplementary Figure S1A). Subsequent PCA analysis showed a similar pattern with a clear separation of treatment and control samples in both conditions (Figures 1B,C) with CAN-2409 treatment explaining 87.8 and 89.9% of the sample clustering *in vitro* and *in vivo, respectively.*

KEGG pathway analysis for genes with fold changes >0 (positive) and < 0 (negative) revealed transcription related pathways among the top 10 downregulated pathways (Figure 1D), while only one pathway –cytokine-cytokine receptor interaction appeared to be upregulated *in vitro* (data not shown). *In vivo*, we also observed a downregulation of transcription associated pathways, with an additional and highly significant downregulation of the KEGG pathway “cell cycle.” Among the top 10 upregulated pathways *in vivo* were cell signaling and cell–cell communication pathways (Figure 1D).

### 3.2. Significant downregulation of p53 pathway after CAN-2409 treatment

**Figure 2:**
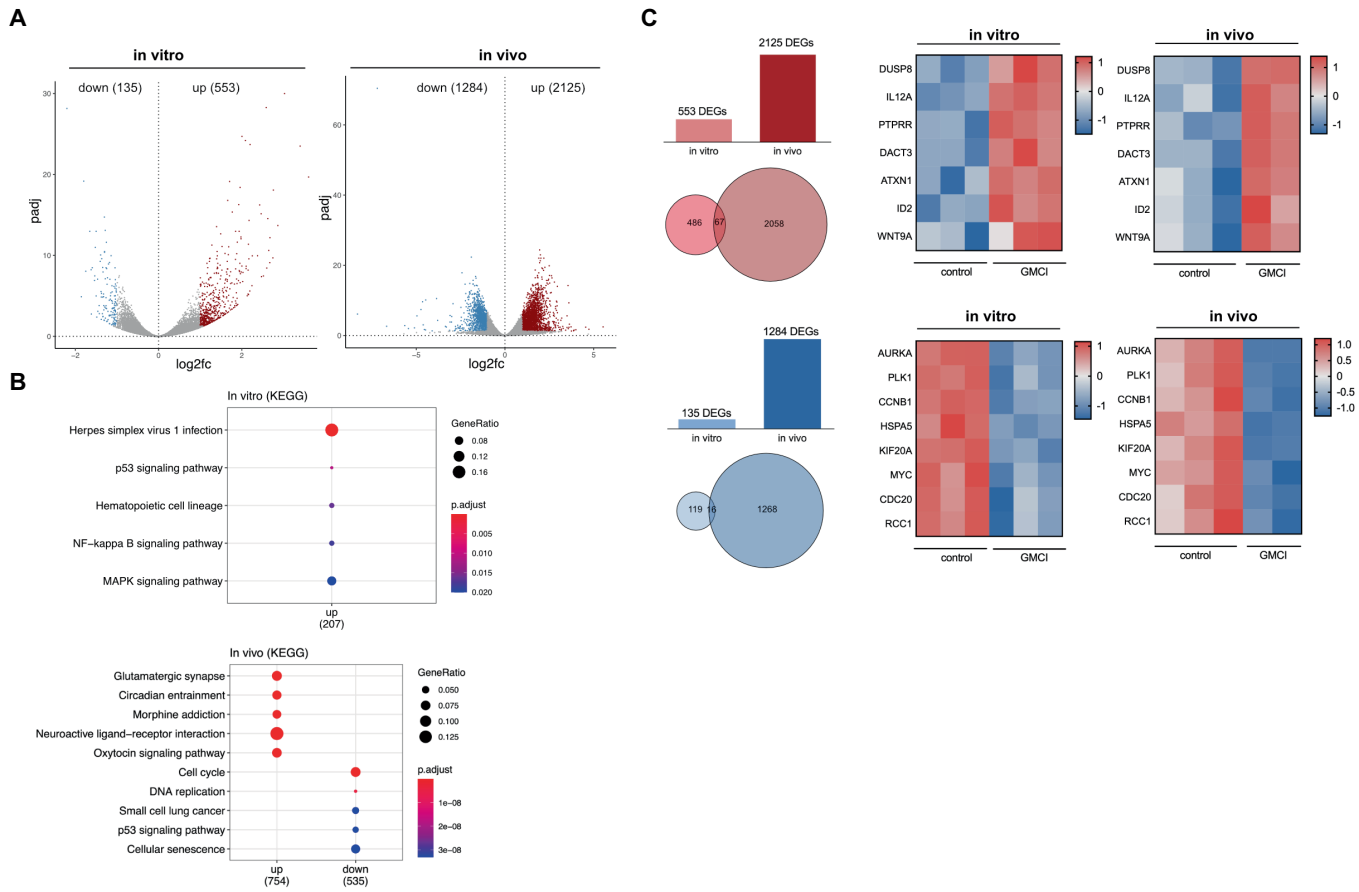
**(A)** Distribution of DEGs *in vitro* and *in vivo* After applying filter criteria (*p* < 0.05, log2FC > 1/< −1) we detected 135 downregulated and 553 upregulated DEGs *in vitro* and 1,284 downregulated and 2,125 upregulated DEGs *in vivo*. **(B)** TOP 5 KEGG pathways of significant DEGs *in vitro* and *in vivo* No downregulated KEGG pathways were detected *in vitro*, while an enrichment, i.e., for “p53 signaling” and “MAPK signaling” was observed. *In vivo*, among others downregulation of “Cell cycle,” “DNA replication” and “p53 signaling.” **(C)** Comparison of significant DEGs *in vitro* and *in vivo* 67 common upregulated and 16 downregulated genes were detected, a relevant selection is displayed in adjacent heatmaps.

To narrow down the extensive CAN-2409 associated effects on the transcriptome, both datasets were further filtered. For any subsequent analyses, only DEGs with an adjusted value of *p* <0.05 were considered and then divided into upregulated (log2 fold change >1) and downregulated (log2 fold change <1). *In vitro*, we detected a total of 135 downregulated and 553 upregulated genes; *in vivo*, 1,284 downregulated and 2,125 upregulated genes were observed (Figure 2A).

To track differential pathway enrichment, we applied a second KEGG pathway analysis only with the significant DEGs as mentioned above. *In vitro*, we observed an upregulation of the KEGG pathways “Herpes simplex virus infection,” “p53 signaling,” “NFkB signaling” and “MAPK signaling,” while no downregulated pathways were detected. *In vivo*, KEGG pathways “cell cycle,” “DNA replication,” “small cell lung cancer,” “p53 signaling” and “cellular senescence” scored highest among the downregulated, while the upregulated pathways were dominated by “Glutamatergic synapse,” “circadian entrainment,” “morphine addiction,” “neuroactive ligand-receptor interaction” and “oxytocin signaling” (Figure 2B).

Although KEGG pathway enrichment analysis revealed converse regulation for the p53 pathway *in vitro* and *in vivo*, strategically important genes that participate in p53 signaling, but also regulate cell cycle progression show similar dynamics: among the 16 commonly downregulated genes were *AURKA*, *MYK*, *CCNB1*, *PLK1*, *HSPA5*, *RCC1* and *CDC20*. Within the 67 commonly upregulated genes, we detected *DUSP8*, *PTPRR*, *DACT3*, *ATXN1*, *ID2*, *WNT9a* and the proinflammatory cytokine *IL12a* (Figure 2C).

### 3.3. CAN-2409 halts mitotic progression and induces apoptosis

**Figure 3:**
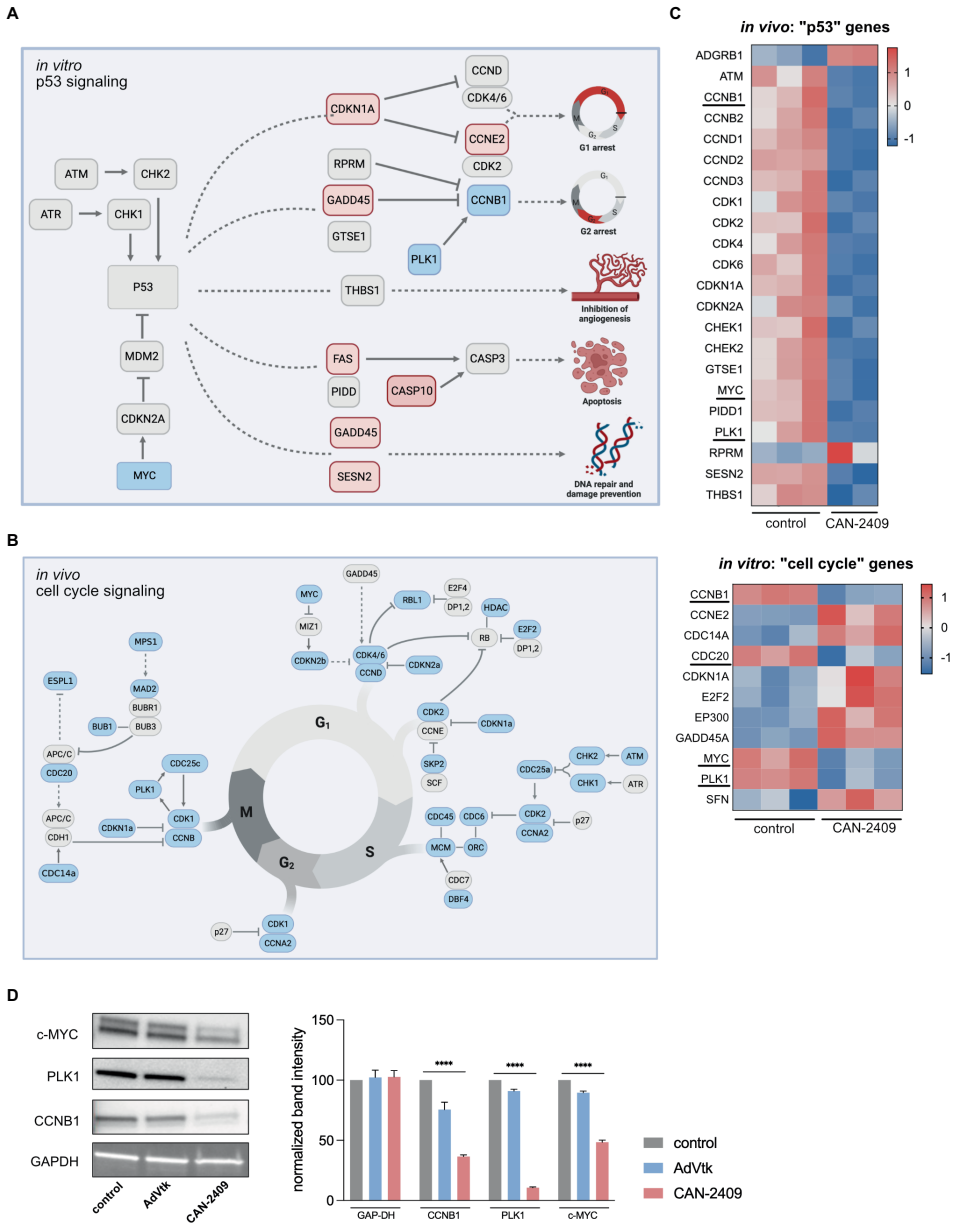
**(A)** Enrichment of p53 signaling pathway *in vitro* Key players of p53 signaling are upregulated including *CDKN1A*, *GADD45* and *FAS*, while *MYC* is downregulated, culminating in G1/2 arrest, increased apoptosis and DNA repair. Modified after KEGG pathway “p53 signaling.” **(B)** Reduction of cell signaling pathway *in vivo* We observed a global downregulation of cell cycle related genes *in vivo*, including checkpoint modulators of G_1_/S-phase (*CDK4/6-CCND*), S/G2-phase (*CDK2-CCNA*) and G_2_/M-phase (*CDK1/CCNB*), pointing towards reduced mitotic activity. **(C)** Heatmaps of p53 signaling related genes *in vivo* and cell cycle related genes *in vitro* Compared to *in vitro*, the majority of p53 signaling related genes is downregulated after treatment with CAN-2409. A similar dynamic under both conditions is displayed for *PLK1*, *CCNB1* and *MYC* which are part of both p53 and cell cycle signaling. Commonly downregulated genes are highlighted. **(D)** Immunoblots of relevant cell cycle targets. Immunoblots of G9_pCDH after 48 h incubation with CAN-2409 show reduced protein abundance of CCNB1, PLK1, and c-MYC compared to control. Multiple unpaired t-tests of normalized band intensities showed significance compared to control (CCNB1, PLK1, and c-MYC *p* < 0.000001). *N* = 1, triple measurement of band signal intensity.

As outlined, CAN-2409 is associated with significant alterations of key molecules of the p53 signaling pathway *in vitro*, including *CDKN1a* (triggering G1), *GADD45* (blocking progression into S-phase), *FAS* and *CASP10* (inducing apoptosis) (Figure 3A).

*In vivo*, KEGG pathway “Cell cycle” was significantly enriched, with a broad downregulation of genes participating in this pathway noted, including checkpoint modulators at the transition from G_1_ to S-phase (*CDK4*/*6*-*CCND*), S to G2-phase (*CDK2*-*CCNA*) and G_2_ to M-phase (*CDK1*/*CCNB*), suggesting a general decrease in mitotic activity (Figure 3B). While cyclin-dependent kinases are not differentially expressed, a reduced expression of central cell cycle regulators like *CCNB1*, *CDC20*, *PLK1,* and *MYC* was observed *in vitro,* as those genes are also part of p53 signaling (Figure 3C); moreover, we also observed upregulation of *CDKN1A* and *GADD45* -both inhibitors of cell cycle progression which contribute to cell cycle arrest in G_1_ and S-phase. Although CAN-2409 is associated with different nuances of pathway enrichment under both conditions, common strategic genes are similarly altered – namely *CCNB1*, *PLK1* and *MYC* (Figure 3C), pointing towards similar transcriptome modifications *in vitro* and *in vivo*, which was confirmed on the protein level as well (Figure 3D), clearly demonstrating reduced expression of CCNB1, PLK1 and MYC after treatment with CAN-2409 compared to control or AdVtk alone. These findings were confirmed in another GSC cell line (G98) with reduced expression of PLK1 and CCNB1 after CAN-2409 compared to control, while MYC was not altered (Supplementary Figure S2).

### 3.4. CAN-2409 leads to significantly increased expression of IL-12A *in vitro* and *in vivo*

Glioblastoma exhibits a distinct cytokine profile with certain pro-tumorigenic cytokines contributing to its malignant features such as IL-6, IL-10 and TGF-β, while others are expressed at lower levels and may have tumor suppressing properties, e.g., IL-4 and IL-12 (11).

**Figure 4:**
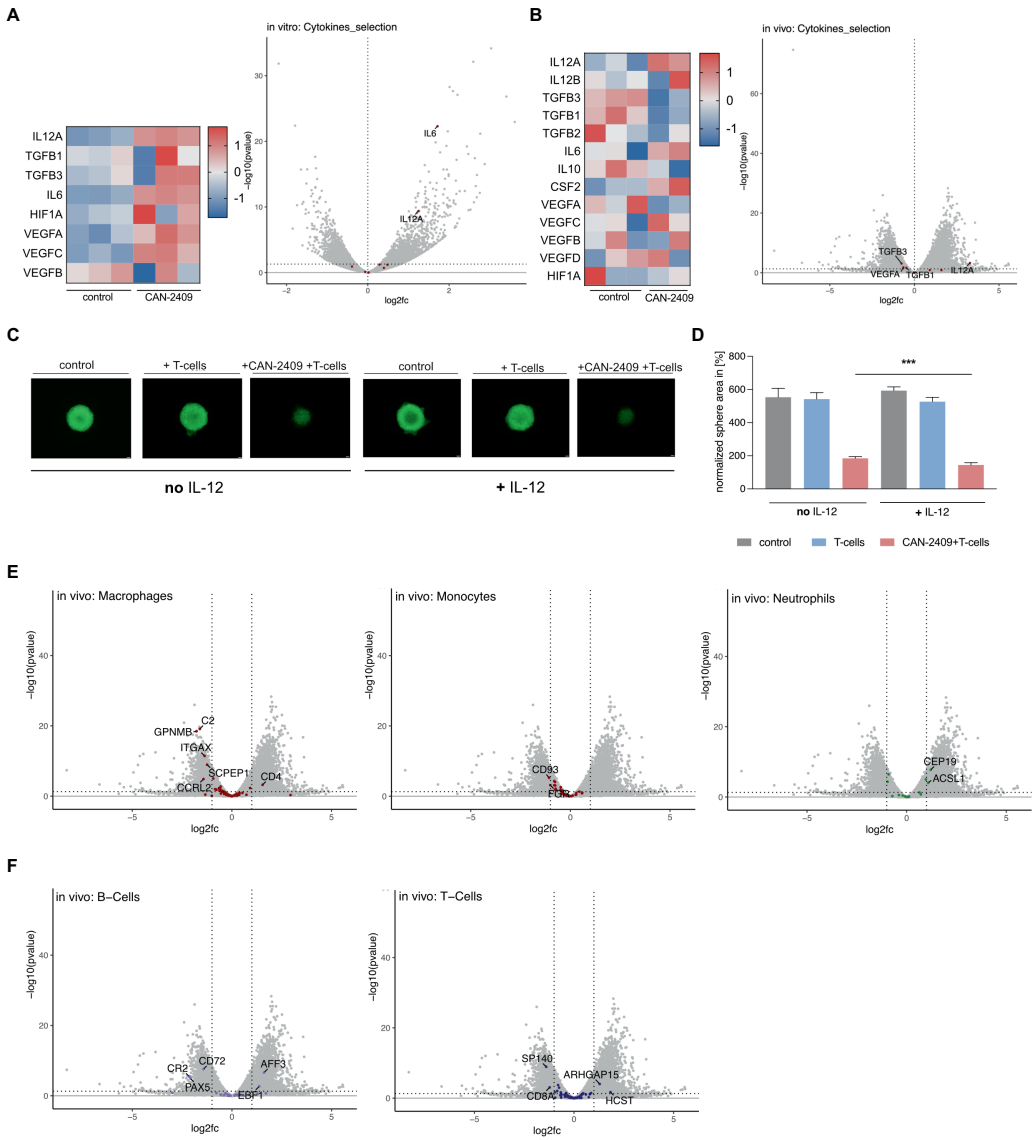
**(A)** Cytokine gene expression *in vitro* Increased expression of *IL12A*, *TGFB*, *IL6*, *HIF1A* and *VEGF* after treatment with CAN-2409 compared to control (heatmap). Adjacent Volcano plot demonstrating significant increased expression for only *IL12A* and *IL6* (cut off: p < 0.05, log2(fc) > 1/<−1). **(B)** Cytokine expression *in vivo* Increased expression of *IL12A*, partly *IL12B*, *IL6*, *CSF2* and partly of *VEGF* as well as decreased expression of *TGFB*, *IL10* and *HIF1A* after treatment with CAN-2409 compared to control (heatmap). Adjacent Volcano plot showing significant increased expression for *IL12A* and decreased expression for *VEGF* and *TGFB1/3* (cut off: *p* < 0.05, log2(fc) > 1/<−1). **(C,D)** Efficiency of *T*-cell killing by CAN-2409 is enhanced by concomitant IL-12 *T*-cell killing assays with G9_pCDH (green) showed significant reductions of the tumor sphere area under the combination treatment with IL-12 and CAN-2409 compared to the same treatment without IL-12 (*p* = 0.0008 – *p* = 0.0728) scale bar = 500 μm. *N* = 3, exemplary images and graph. **(E)** Diminished macrophage/monocyte signature after CAN-2409 treatment *in vivo* Volcano plots of macrophage, monocyte and neutrophil associated genes showing significant decreased expression after treatment with CAN-2409; for neutrophils an enhanced expression of neutrophil associated genes *CEP19* and *ACSL1* in accordance with Nirmal et al. (12). **(F)** Differential regulation of B-and T-cell associated genes *in vivo* For B-cells a diminished expression for *CR2*, *CD72* and *PAX5* with concomitant heightened expression of *AFF3* and *EBF1* was noticed. Interestingly, in T-cell signature analysis *CD8a* together with *SP140* were downregulated, whereas *ARHGAP15* and *HCST* were upregulated.

*In vitro*, we observed an upregulation of *IL12A*, *IL6*, and *VEGFC*, while no clear pattern for *TGFß*, *HIF1a* and *VEGFA/B* could be defined; significant differential expression could only be noted for *IL12A* and *IL6* (Figure 4A). As the *in vitro* samples are lacking the influence of immune cells, we further analyzed the *in vivo* datasets for a more accurate depiction of cytokine pattern changes, as these include not only tumor mass, but also infiltrating immune cells. Here, we observed -in animals treated with CAN-2409 -an upregulation of the anti-tumorigenic cytokine related genes *IL12A* (significant: log2FC 3.279160537, value of *p* 0.00054542, adj value of p 0.002450255) and *IL12B* (not significant), but also of pro-tumorigenic related genes for *IL6*, *CSF2* and *VEGF*. However, the majority of pro-tumorigenic associated genes including *TGFß*, *IL10*, *VEGFA* and *HIF1A* were downregulated (Figure 4B). Similar to the *in vitro* dataset, only *IL12A* appeared to be significantly overexpressed.

CAN-2409 has been shown to induce a Th1-like immune response *in vivo*, including increased production of IL-12 (13). While we observed differential expression of cytokine regulating genes under both conditions, *IL12A*, encoding for a subunit of IL-12 (14), was stably overexpressed *in vitro* and *in vivo*. *In vivo*, we also observed an (although not significantly) increased expression of *IL12B;* for successful translation of the cytokine IL-12 on protein level, both *IL12A* and *IL12B* mRNA transcripts are necessary, therefore one can assume that in contrast to the *in vitro* data, above outlined *in vivo* expression patterns are biologically relevant, i.e., leading to increased IL-12 production on the protein level. Moreover, the concordant expression dynamics of *IL12A* under both conditions point towards *IL12A* being a central molecule for CAN-2409’s mode of action. To confirm the importance of this cytokine for CAN-2409’s therapeutic efficiency, we validated the effect of IL-12 and CAN-2409 in T-cell killing assays in the presence and absence of recombinant human IL-12 (Figure 4C). Statistical analysis on day 6 of co-culture with CD8^+^ T cells showed a significant reduction of the tumor sphere area under the combination treatment with IL-12 compared to the same treatment without IL-12 in all cell lines (G9_pCDH, *p* = 0.0008**** –0.0728 ns, *n* = 3) under co-culture with IL-12 (Figure 4D). This suggests that indeed the efficacy of CAN-2409 is enhanced by IL-12.

### 3.5. Decreased expression of macrophage and monocyte gene signatures after CAN-2409 *in vivo*

The characteristics of glioblastoma are to a certain extent defined by the interaction of tumor cells and the surrounding tumor microenvironment; especially macrophages and monocytes – which account for up to 50% of the tumor mass (15) and contribute to the malignant features of glioblastoma. To investigate CAN-2409’s effect on the tumor microenvironment’s transcriptome, we analyzed our *in vivo* data for expression of immune cell signatures according to (12) (detailed tables in Supplementary material S2). We observed significantly reduced expression of myeloid signature genes, whereas we saw the opposite for neutrophils and no distinct dynamics for B-cell or T-cell cell related genes.

The comparison with previously described monocyte and macrophage gene sets (12) revealed overexpression of *CD4*, whereas the majority of genes detected were either not altered or significantly downregulated, like inflammation associated *C2* (complement protein), *GPNMB* (involved in inflammation (16), associated with promoting CSC features (17)), *ITGAX* (encoding CD11c surface protein (18)), *SCPEP1* and *CCRL2* (altering M1/2 polarization (19), promoting T-cell mediated anti-tumor immunity (20))(macrophages) and *CD93* (general myeloid marker (21)) and *FGR* (protooncogene, normal myeloid marker (22))(monocytes). For neutrophils, upregulation of *CEP19* and *ASCL1* was observed (Figure 4E).

In our previous studies of tumor cellular composition by mass cytometry, B-cells were shown to be increased upon treatment with CAN-2409 (23). Compared to above mentioned datasets, a decrease of B-cell related genes *CD72*, *PAX5* and *CR2* (24–26) was noticed, while *AFF3* and *EBP1* were upregulated. CAN-2409’s immune cell related effects are mediated *via* CD8^+^ *T*-cells, but also an increase in Tregs was reported previously (27); here we observed a downregulation of T-cell related genes *SP140* and *CD8a* and an increase of *ARHGAP15* and *HCST* (Figure 4F). Thus our *in vivo* studies show molecular transcriptomic alterations indicative of changes in the tumor microenvironment in response to CAN-2409 treatment.

## 4. Discussion

CAN-2409 has been shown to induce immunogenic cell death in glioblastoma and transform the immunologically “cold” into a “hot” tumor microenvironment. Although this is achieved by interfering with tumor cell DNA through incorporation of an antimetabolite -the HSV-tk metabolized prodrug ganciclovir, acyclovir or valacyclovir. The resulting alterations of the tumor transcriptome have not been assessed. CAN-2409 has been thoroughly characterized *in vitro* and *in vivo*, especially regarding it immunological implications in previous publications (13, 27, 28). We conducted an RNA-Seq analysis of both *in vitro* and *in vivo* samples to characterize the CAN-2409 mediated transcriptional modifications and further alterations caused by interaction with the tumor microenvironment.

In the current study, we showed that CAN-2409 is associated with a downregulation of transcriptionally relevant pathways under both conditions. More detailed analysis revealed a specific *in vitro* downregulation of p53 signaling and *in vivo* downregulation of cell cycle signaling, with reduced expression of *MYC*, *CCNB1*, *CDC20* and *PLK1* in both conditions. Analyses for cytokine expression profiles indicated upregulation of *IL12a in vitro* and *in vivo,* with *IL12b* showing increased expression *in vivo*, although not being significantly overexpressed. Immune cell signature profiling with the *in vivo* data pointed towards diminished expression of macrophage and monocyte relevant genes.

Ganciclovir, which together with CAN-2409 (Ad.HSV-tk) constitutes the CAN-2409 therapeutic regimen, inflicts DNA damage, culminating in activation of ATR and ATM (29). The latter are involved in DNA damage control and hence mitotic regulation (30). ATR inhibition further increased CAN-2409’s tumor cell killing abilities (23).

The HSV-tk/ganciclovir gene suicide system was shown to be associated with cell cycle arrest in the G1/S-phase: Zeng et al. reported that after treatment of a breast cancer cell line with a Tet-on-HSV-tk/GCV system, an increment of the *S*-phase status was noticed along with increased expression of *CDKN1a* and decreased expression of *CDK1*, *CCNB* and *PCNA*, suggesting that HSV-tk/GCV’s modus operandi includes cell cycle inhibition (31). These reports are complemented with our findings: *in vitro*, an overexpression of *CDKN1a* as well as decreased expression of *CCNB1* (which was also the case for the *in vivo* data) was observed. Whereas *CDK1* was not significantly altered *in vitro*, its expression was diminished *in vivo*. While we noticed some discrepancies in expression between *in vitro* and *in vivo*, cell cycle and p53 relevant genes, i.e., *CCNB1*, *PLK1*, *MYC*, *CDC20* were similarly downregulated in both conditions, supporting the hypothesis that indeed tumor cell killing features of this gene suicide system are induced *via* disruption of the cell cycle, which also translated onto the protein level.PLK1, which phosphorylates CCNB1 (32), is overexpressed in several cancers (33). PLK1 depletion as well as CCNB1 silencing have been reported to mediate apoptosis and G2/M arrest (34–36). Inhibition of PLK1 combined with radiotherapy (37) or temozolomide chemotherapy (38) increased the efficiency of those treatments in glioblastoma models.

As previously shown, CAN-2409 induces a Th1-like immune response, i.e., increased expression of IL-12, IL-2, IFN-γ, TNF-α, and GM-CSF (13). IL-12 specifically is considered as an anti-tumorigenic cytokine in various malignant diseases (39). Kerkar et al. reported that the tumor microenvironment was considerably altered towards inflammation by *in vitro* activated IL-12 producing CD8^+^ *T*-cells (40). Current strategies to further enhance previously established immunotherapies for glioma include intratumoral delivery or increase of IL-12 (41–43). Here, we show an increase of *IL12a in vitro* and *IL12a* and *IL12b in vivo*. IL-12 consists out of two subunits, light chain p35 (encoded by *IL12a*) and heavy chain p40 (encoded by *IL12b*) (14). For a physiologically functioning IL-12, both subunits are necessary. While we found upregulation of *IL12a in vitro*, we observed upregulation for both *IL12a* and *IL12b in vivo*, i.e., the latter is presumptively associated with increased biologically active cytokine expression of IL-12 which has been previously reported by Vile et al. (13). We also showed that upregulation of IL-12 is an integral part of CAN-2409’s mode of action (Figures 4A–D). Indeed treatment with CAN-2409 under addition of recombinant IL-12 increased tumor cell killing abilities in presence of activated *T*-cells significantly. IL-12 therefore might serve as a potential biomarker in patients treated with CAN-2409.

The glioblastoma microenvironment is characterized by a large proportion of myeloid cells, such as macrophages. Tumor associated macrophages (TAMs) contribute to the malignant features of glioblastoma by secreting pro-tumorigenic factors, e.g., IL-10 and TGF-*β* (44). Treatment with CAN-2409 has been shown to reduce the number of TAMs, which can be reversed by concomitant treatment with dexamethasone (28) or enhanced by parallel inhibition of the ATR-CHK1 axis (23). RNA Seq analyses for immune cell profiles confirm previous observations on a transcriptomic level, as we demonstrated a reduction of macrophage/monocyte related genes after treatment with CAN-2409 (Figure 4E).

Taken together our findings show that CAN-2409 broadly changes the tumor cell transcriptome -which is further modulated by the interplay with the tumor microenvironment. Our observations suggest that the CAN-2409 induced transcriptomic alterations make a major contribution to the treatment’s tumor cell killing abilities and complements its immunogenic impact.

## Data availability statement

The original contributions presented in the study are publicly available. This data can be found under GEO accession number GSE230661 (https://www.ncbi.nlm.nih.gov/geo/query/acc.cgi?acc=GSE230661).

## Ethics statement

The animal study was reviewed and approved by IACUC Brigham and Women's Hospital Boston, United States.

## Author contributions

SL, MK, and EC: study conceptualization, design, and project administration. MK, MN, MZ, ZH, and MH: methodology. MK and SL: investigation and writing–original draft. MK, ZH, and MH: data analysis. SL, EC, and GT: funding acquisition. SL and ZH: supervision. MK, SL, EC, MZ, MN, EC, FB, PT, MH, ZH, and GT: writing–review and editing. All authors contributed to the article and approved the submitted version.

## Funding

This study was funded by NCI P01CA069246. This research was partially funded by NCI R50 CA243706-02 (MN) and Else Kröner Fresenius Forschungskolleg (2015_Kolleg_14) 9 (GT).

## Conflict of interest

FB and PT are employees of Candel Therapeutics.

The remaining author declares that the research was conducted in the absence of any commercial or financial relationships that could be construed as a potential conflict of interest.

## Publisher’s note

All claims expressed in this article are solely those of the authors and do not necessarily represent those of their affiliated organizations, or those of the publisher, the editors and the reviewers. Any product that may be evaluated in this article, or claim that may be made by its manufacturer, is not guaranteed or endorsed by the publisher.
