# Supplementary figures and images for: Uncovering transcriptomic landscape alterations of CAN-2409 in in vitro and in vivo glioma models
Source: Front Med (Lausanne). 2023 May 9;10:1140352. doi: 10.3389/fmed.2023.1140352 (PMC10203593; doi:10.3389/fmed.2023.1140352)

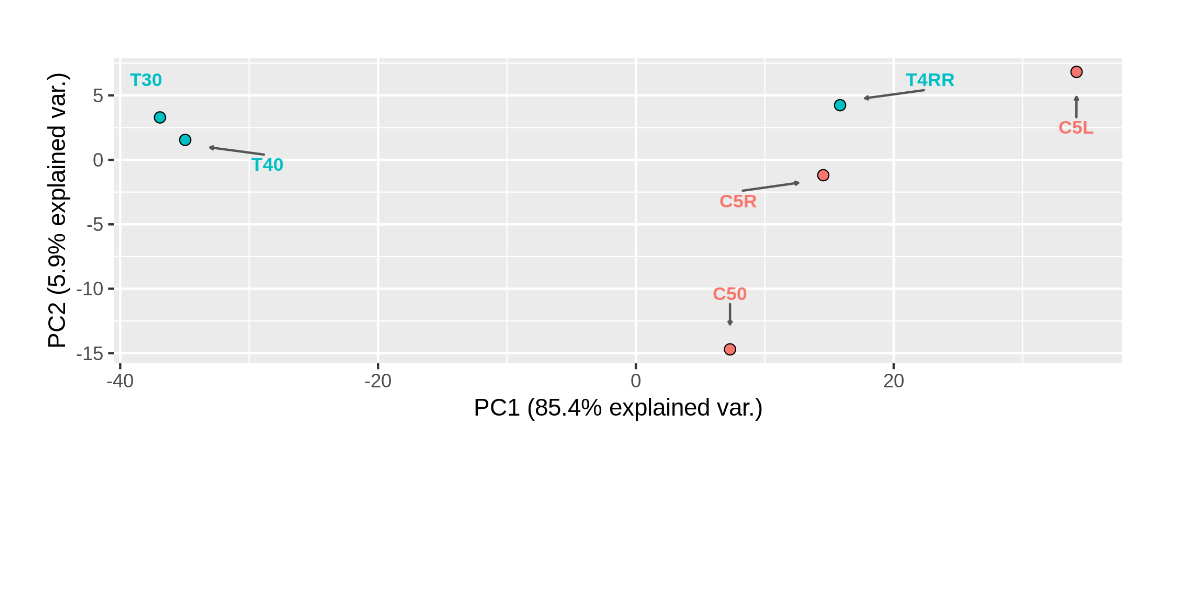

Supplement: Supplementary file 1 [file Image_1.TIFF]

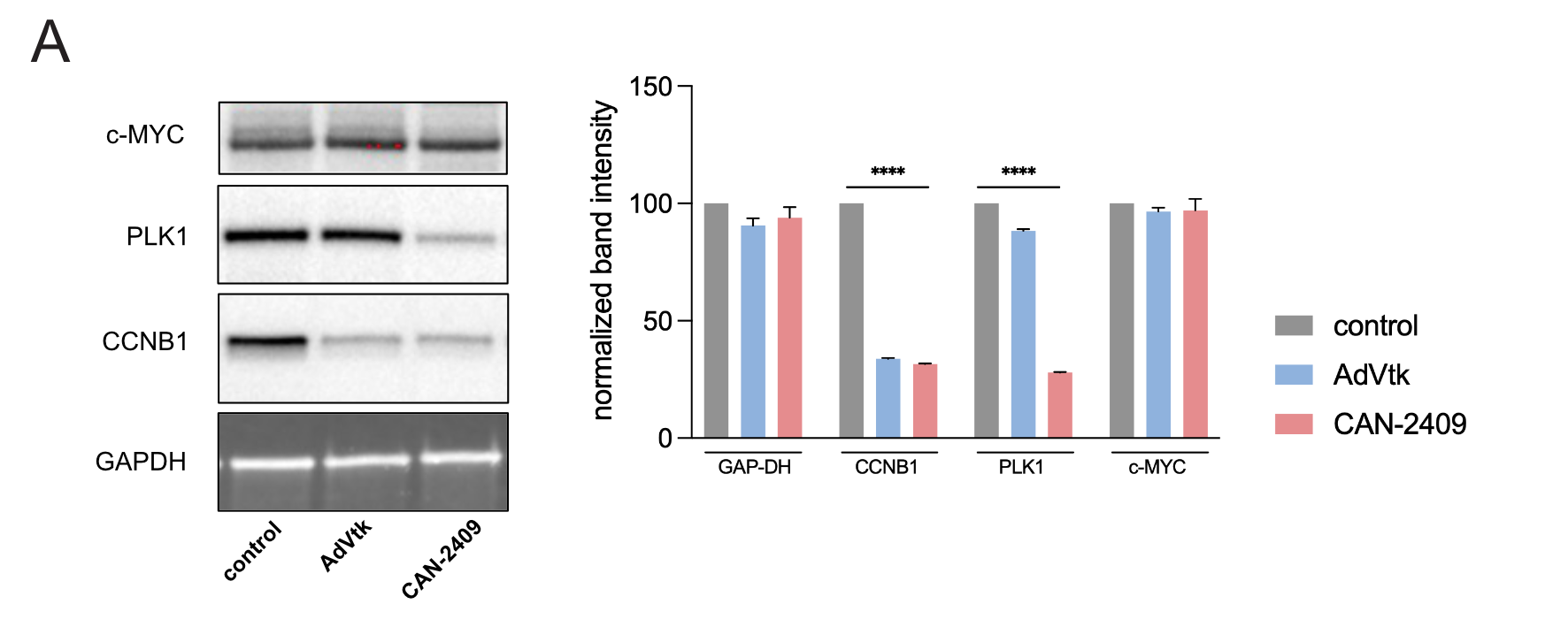

Supplement: Supplementary file 2 [file Image_2.TIFF]
